# High-performance electromagnetic wave absorption in cobalt sulfide flower-like nanospheres

**DOI:** 10.1039/d2ra04764k

**Published:** 2022-09-07

**Authors:** Hao Yuan, Zhidong Liu, Yani Zhang, Jinfeng Ding, Yuping Sun, Min Zhang, Shugang Tan

**Affiliations:** School of Physics and Optoelectronic Engineering, Shandong University of Technology Zibo 255000 People's Republic of China tanshugang@sdut.edu.cn; Anhui Province Key Laboratory of Pollutant Sensitive Materials and Environmental Remediation, School of Physics and Electronic Information, Huaibei Normal University Huaibei 235000 People’s Republic of China zmin@mail.ustc.edu.cn

## Abstract

A heterophase cobalt sulfide absorbing material with petal-like surface structure was prepared by a simple hydrothermal method. The cobalt sulfide sample with the optimal microwave absorption capacity was achieved through regulating the reaction temperature. By regulating the reaction temperature to 200 °C, the optimal reflection loss was −48.4 dB at 16.8 GHz with filler loading of 50%, and the effective absorption bandwidth was 4.3 GHz at Ku band corresponding to a thickness of only 1.5 mm. The petal-like surface structure of cobalt sulfide gradually disappears as the reaction temperature rises, and the reduction of specific surface area has a negative effect on the microwave absorption capacity of the sample. Meanwhile, by adjusting the sample thickness from 1.5 to 5.0 mm, the effective absorption bandwidth could cover almost the whole test frequency range. The results show that the cobalt sulfide absorbing material with regulated reaction temperature has a strong electromagnetic wave absorption ability, light weight, thin thickness and simple synthesis, which is a promising microwave absorbing material for actual application.

## Abstract 2

A heterophase cobalt sulfide absorbing material with petal-like surface structure was prepared by a simple hydrothermal method.

## Graphical abstract

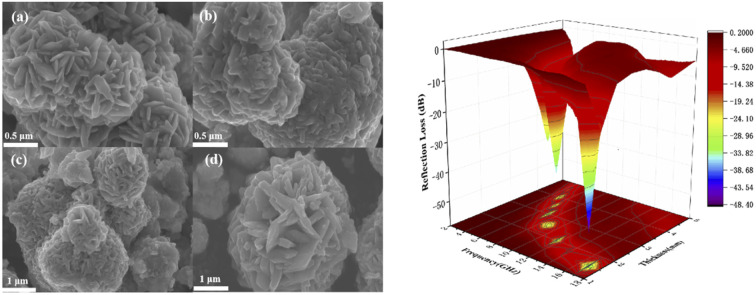

## Introduction

In recent years, electromagnetic wave absorbing materials, which can convert electromagnetic energy into heat energy, have received increasingly attracting attention due to the serious electromagnetic pollution problems caused by the continuous development of electronic information technology and wireless communication means.^1–5^ Generally, the excellent absorbing materials should have the advantages of thin thickness, light weight, wide absorption bandwidth and strong absorption ability. Conventional ones such as carbon nanomaterials, ceramics, ferrite and metal magnets are widely used for electromagnetic wave absorption, but their impedance matching as well as microwave absorption performance is not satisfactory.^6–10^ Researchers have focused on the synergistic effect of multi-component wave absorbing materials.^11–13^ For example, a series of studies have been conducted on the composite of magnetic ferrite materials with two-dimensional dielectric materials, including CoFe_2_O_4_/MoS_2_,^14^ RGO/NiFe_2_O_4_,^15^ RGO/MnFe_2_O_4_ (ref. 16) and ZnFe_2_O_4_/MoS_2_.^17^ However, multi-component composites are usually complicated to synthesize and the ratio regulation between different components is difficult to control. At the same time, multi-component materials are difficult to achieve the goal of lightweight wave absorbing materials. Therefore, improving the impedance matching and absorbing ability of single-component absorbing materials is still a topic worthy of attention.

Semiconductor transition metal sulfides have become a hot research topic in recent years due to its high dielectric properties, low cost and high theoretical capacity. Semiconductor transition metal sulfides, are widely used in photocatalysis, solar cells, lithium batteries and energy storage.^18–20^ Meanwhile, semiconductor metal sulfides exhibit excellent performance in the field of wave absorbing materials due to their strong dielectric loss capability, such as MoS_2_,^21^ CdS,^22^ CuS (ref. 23) and PbS.^24^ In contrast, cobalt sulfide has not attracted sufficient attention because of its stoichiometric ratio diversity, such as Co_9_S_8_,^25^ CoS,^26^ Co_3_S_4_ (ref. 27) and CoS_2_.^28^ In recent years, various morphologies of cobalt sulfide samples have been investigated, such as nanospheres,^29^ nanowires,^30^ nanotubes,^27^ nanosheets,^31^ and flower-like nanocrystals,^32^ which have been shown to optimize reflection loss due to their morphological structural characteristics. However, studies on cobalt sulfide have usually focused on the composite of cobalt sulfide. For example, Huang *et al.* reported the CoS hollow spheres with flower-like morphology by cetyltrimethyl ammonium bromide (CTAB) as surface activator, and the minimum reflection loss of −43.6 dB at 15.6 GHz, while the effective absorption bandwidth is 4.6 GHz at the thickness of 2.0 mm.^33^ A nanoarchitecture of multiwalled carbon nanotubes anchored with CoS nanoplates were synthesized by Huang *et al.*, and the optimal reflection loss was approach to −56.1 dB at 6.6 GHz, but the thickness of the sample increased to 3.6 mm.^34^ It is well known that nanostructured wave absorbing materials in various forms have been prepared. For example, molybdenum disulfide is mostly in the form of nanosheets with a layer-like structure.^35–37^ In contrast, the cobalt sulfide prepared in the form of flower-like nanospheres possesses a large specific surface area and abundant folds, which facilitate the introduction of interfaces and more polarized charges. The spherical overall morphology can also be used to limit the size of the nanospheres using, for example, silica templates, which is also meaningful for the enhancement of the wave absorption properties.^38^ At the same time, heterogeneous materials have unique crystalline phases as well as crystal structures, which may contribute to the improvement of wave absorption properties.^39,40^ Therefore, it is still a challenge to prepare thinner and lighter single-component heterophase cobalt sulfide materials while maintaining the outstanding wave absorption capability.

Herein, we report a simple hydrothermal method for the preparation of heterophase cobalt sulfide single-component wave-absorbing materials with a phase structure work. *Via* regulating the reaction temperature to 200 °C, the optimal reflection loss was −48.4 dB at 16.8 GHz with filler loading of 50%, and the effective absorption bandwidth was 4.3 GHz at Ku band corresponding to a thickness of only 1.5 mm. This cobalt sulfide sample exhibits excellent wave absorption properties with high specific surface area, high electrical conductivity and excellent impedance matching. This study may provide a new idea to further improve the absorption performance of single-component wave-absorbing materials.

## Experimental details

### Materials

Thioacetamide (C_2_H_5_NS, 98%), cobalt chloride hexahydrate (CoCl_2_·6H_2_O, 99%), ethanol (99.7%) and *N*,*N*-dimethylformamidel (DMF, C_3_H_7_NO, 99.5%) were purchased from Sinopharm Chemical Reagent, China. All chemical reagents in this study were used without any further purification.

### Synthesis of cobalt sulfide nanoparticles

0.8 g of C_2_H_5_NS and 0.4 g CoCl_2_·6H_2_O were dispersed into 14 mL of DMF/water mixture soultion (1 : 1 v/v). The mixed precursor solution was stirred for 30 min at room temperature by using a magnetic stirrer. Transferring the mixed solution to a 50 mL Teflon-lined stainless steel autoclave and maintained at 200 °C for 24 h. The precursor solution was centrifuged at 8000 rpm when it naturally cooled to room temperature. The precipitates were conducted by centrifugation, washed with DI water and ethanol, and dried at 60 °C under vacuum for 12 h. The product as C1, which obtained after drying. Different reaction temperatures of the product as C2, C3, and C4 were corresponding to 210 °C, 220 °C and 230 °C, respectively.

### Material characterizations

The crystal structures of the samples were determined by powder X-ray diffraction (XRD) on a Bruker Advance D8 X-ray diffractometer using Ni-filtered Cu Kα radiation source (40 kV, 40 mA, *λ* = 1.5418 Å). The microstructure of samples characterized by Field emission scanning electron microscopy (FESEM, SU8200). The parameters of permeability and permittivity in 2.0–18.0 GHz of the samples were characterized by using the vector network analyzer (VNA, AV3629D), which based on a transmission/reflection model. Before the parameters of wave absorption performances characterization, the samples with different filling rates of 30 wt%, 40 wt%, 50 wt% were prepared by homogenous mixing the samples and the paraffin wax. Similarly, samples of various filling ratios were measured after uniformly mixing the paraffin. Then the samples were pressed into a cylindrical toroid with an inner diameter of 3.04 mm, an outer diameter of 7.00 mm, and a thickness of about 2.00 mm.

## Results and discussion

**Fig. 1:**
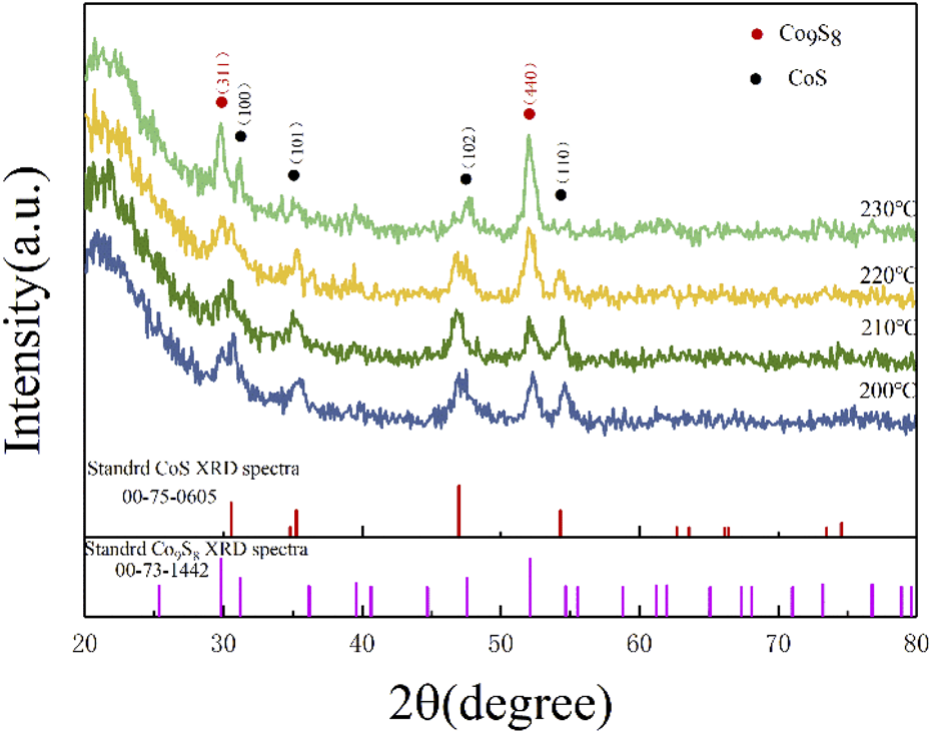
XRD curves of the cobalt sulfide with various of reaction temperature.

As shown in Fig. 1, the XRD patterns and Bragg peaks match well with the CoS (JCPDS Card no. 75-0605) and the Co_9_S_8_ (JCPDS Card no. 73-1442), which implies the successful generation of target products. The four main peaks including (100), (101), (102) and (110) of CoS at around 30.6°, 35.3°, 47.0° and 54.3° were clearly displayed in a changing temperature range. Meanwhile, the Bragg peaks (311) and (400) corresponding to 29.8° and 52.0°, which were the main peaks of Co_9_S_8_, are also shown in Fig. 1. Interestingly, the partial characteristic diffraction peaks intensity of CoS gradually decreases with increasing reaction temperature, and the Bragg peaks of (110) disappear with the reaction temperature of 230 °C, and the other peak intensity also become weaker with increasing reaction temperature. On the contrary, the peak intensity of (311) and (440), which belong to the Co_9_S_8_, were positive correlation with the reaction temperature. It is noteworthy that the relative intensity of the peaks changed considerably with reaction temperature. This may be attributed to a change in the ratio of the two components in the product, with more Co_9_S_8_ phases being generated as the reaction temperature rises. The quantitative analysis of the two phases revealed that the ratio of the two phases is close to 1 : 1 when the temperature is greater than 200 °C, while the content of CoS exceeds that of Co_9_S_8_ when the temperature is less than 200 °C. Similar with previous studies, as the temperature increases, the size of the product gradually increases while the lattice gap becomes larger, leading to a change in the crystalline phase. Therefore, it can be concluded that the reaction temperature affect the crystalline phase of the products to some extent.^41^

**Fig. 2:**
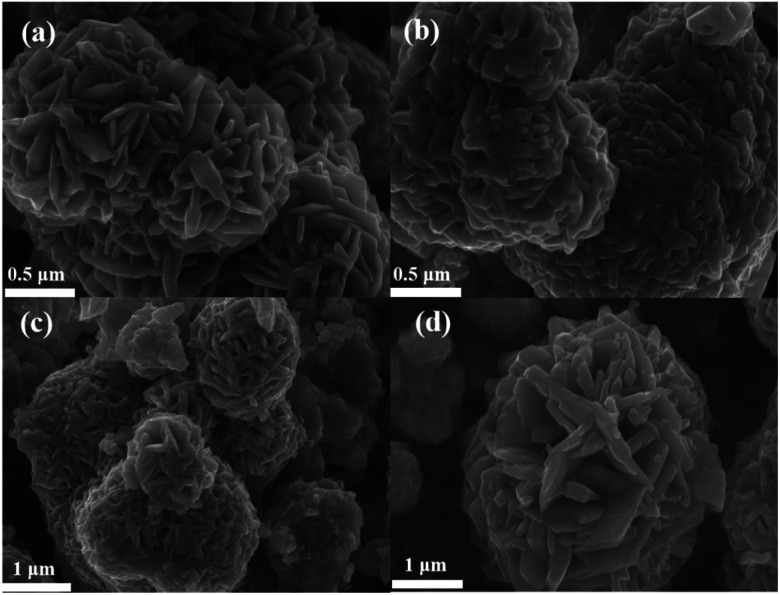
(a) The SEM image of C1 after reacting at 200 °C, (b) the SEM image of C2 after reacting at 210 °C (c) the SEM image of C3 after reacting at 220 °C, the SEM image of C4 after reacting at 230 °C.

**Fig. 3:**
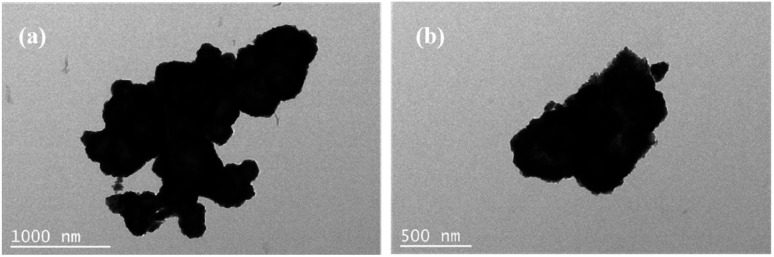
(a) and (b) The TEM image of CoS under different resolutions.

Fig. 2(a) shows the microscopic morphology of sample C1, with more cobalt sulfide particles wrapped around the primary particles as the reaction proceeds, forming a flower-like morphology with a rich interface. This structure with multiple folds on the surface facilitates further absorption of electromagnetic waves. And the cobalt sulfide is easy to form hollow spheres which contributes to the loss of absorbed electromagnetic waves based on the well-known Ostwald ripening process.^42^ The H_2_S bubbles generated by the thermal decomposition of thioacetamide can produce hollow nanostructures. As the reaction proceeds, cobalt sulfide nanoparticles with hollow cavities may be produced based on the previous reports.^33^Fig. 3(a) and (b) characterize the TEM images of the cobalt sulfide samples at different resolutions, respectively. It is confirmed that the cobalt sulfide sample forms a hollow structure with the reaction, which facilitates the absorption of electromagnetic waves.Fig. 2(b)–(d) depict the microscopic morphology of the samples of C2, C3 and C4 at reaction temperatures of 210 °C, 230 °C and 230 °C, respectively. It is noteworthy that the petals on the surface of the C2, C3 and C4 are gradually decreasing compared to C1, which showing a rough and wrinkled appearance, and there is no significant change in nanoparticle size. This phenomenon may indicate that changing the reaction temperature conditions plays an important role in regulating the morphology of nanoparticles. As the reaction temperature increases, the surface of cobalt sulfide produces more Co_9_S_8_ particles adsorbed on the surface of the initial particles in excess of S^2−^, which might be one of the important factors in changing the microstructure of cobalt sulfide. Previous similar studies have shown that hollow materials, polyhedral clusters with rich surfaces and materials with flower-like surfaces have the potential to be excellent wave absorbers.^43–47^ For example, hollow CoNi/C which near the percolation threshold possesses RL min value of −67.22 dB at 1.70 mm.^44^ Cobalt sulfide is synthesized by simple hydrothermal method without template and calcination treatment, which means low cost and easy to enlarge. Meanwhile, cobalt sulfide has a lighter weight in line with the pursuit of lightweighting of wave absorbing materials. According to the conductivity network model and the mechanism of aggregation-induced charge transport, lightweight absorbing materials usually have a high density of micro-conductivity networks, leading to enhanced conductivity loss.

**Fig. 4:**
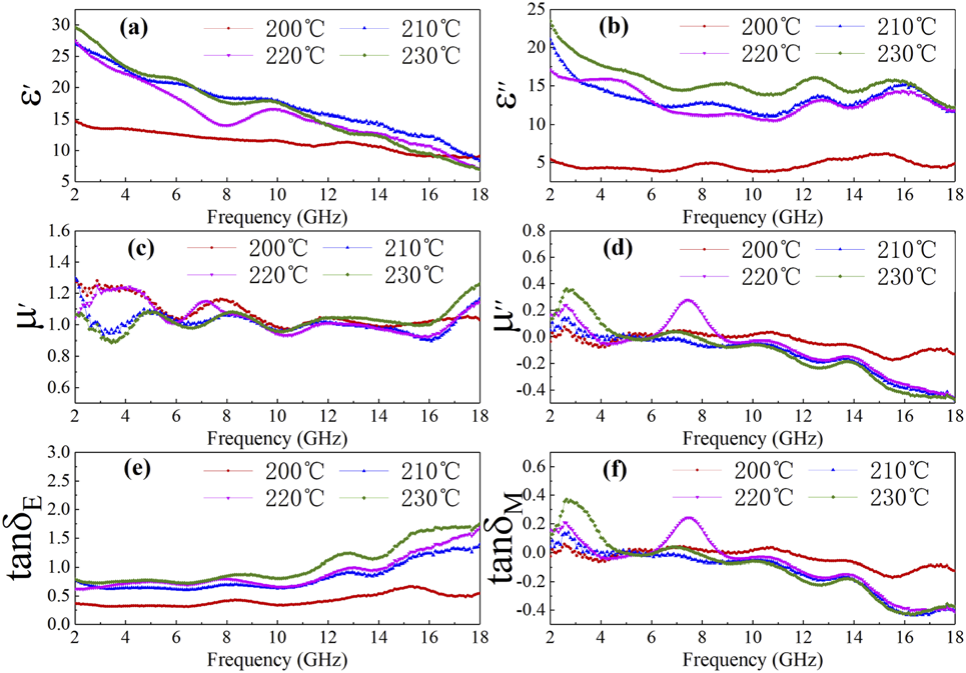
The *ε*′ (a), *ε*′′ (b), *μ*′ (c), *μ*′′ (d), (e) dielectric loss tangent (tan *δ*_e_ = *ε*′′/*ε*′) and (f) magnetic loss tangent (tan *δ*_m_ = *μ*′′/*μ*′) of C1 to C4 paraffin composites with 50% filling ratio.

In order to further investigate the electromagnetic absorption mechanism of cobalt sulfide, the complex permittivity real part (*ε*′), permittivity imaginary part (*ε*′′), permeability real part (*μ*′), and permeability imaginary part (*μ*′′), dielectric loss tangent (tan *δ*_e_ = *ε*′′/*ε*′) and magnetic loss tangent (tan *δ*_m_ = *μ*′′/*μ*′) were tested at the frequency range of 2–18 GHz of all samples. And these electromagnetic parameters are shown corresponding to Fig. 4(a)–(f), respectively. The complex permittivity and the permeability can be expressed as *ε*_r_ = *ε*′ − *jε*′′ and *μ*_r_ = *μ*′ − *jμ*′′. *μ*′ and *ε*′ are the real part of permeability and the real part of dielectric constant of the material respectively, which are a measure the magnetic storage capacity and the electric storage capacity of the material, respectively. And *μ*′′ and *ε*′′ represent the imaginary part of permeability and the imaginary part of dielectric constant of the material, which reflect the strength of the intrinsic magnetic loss and dielectric loss of the material.

As shown in Fig. 4(a), the real part of the dielectric constant is approximately positively correlated with temperature. Similar trend is observed for the imaginary part of the dielectric constant from Fig. 4(b). As displayed in Fig. 4(c) and (d), the real part of magnetic permeability and the imaginary part of magnetic permeability converge to 1 and 0 respectively, which implies that the magnetic loss has little effect on the microwave absorption performance of cobalt sulfide. Fig. 4(e) shows the dielectric loss tangent falling then rising with increasing temperature. And Fig. 4(f) indicates that the magnetic loss tangent variation of cobalt sulfide is small, consistent with the previous analysis. We know that the continuous accumulation of space charge at the interface between paraffin and sample results in the formation of local conductive networks and heterogeneous systems. This phenomenon of electromagnetic parameter change may be due to the continuous generation of cobalt sulfide which changes the interfacial area and conductive network in the paraffin matrix to the extent that it affects the interfacial polarization between cobalt sulfide and paraffin. In summary, the sample with 50% filling ratio may have excellent microwave absorption at 200 °C, which is also proven below.

It is well known that polarization loss and conductivity loss jointly affect dielectric loss, and polarization loss plays a major role in the loss of wave absorbing materials. Polarization is usually classified into ion polarization, electron polarization, dipole polarization and interface polarization. Among them, ion polarization and electron polarization can be neglected in the microwave range (2–18 GHz). Therefore, dipole polarization and interfacial polarization can be considered as the main sources of dielectric losses in absorbing materials. However, the dipole loss may mainly originate from the redistribution of charges due to heterogeneous cobalt sulfide particles, which form intrinsic dipoles and defective dipoles. And the abundant defects can be regarded as the polarization center, which contributes to the polarization loss to a certain extent and helps to improve the electromagnetic wave absorption capacity.^48^ Based on the Debye theory, *ε*′, *ε*′′ and the relationship between *ε*′ and *ε*′′ can be expressed as:^49^1
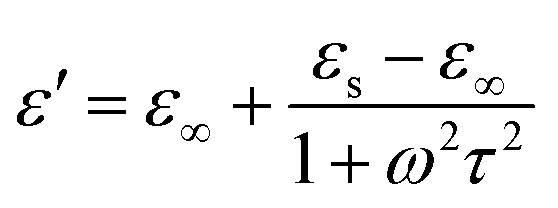
2
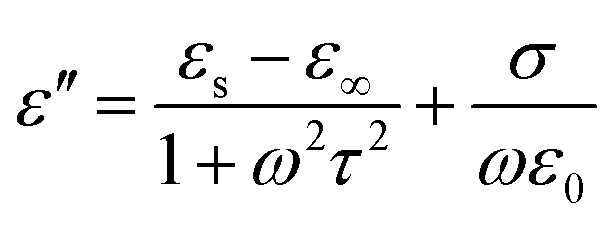
3
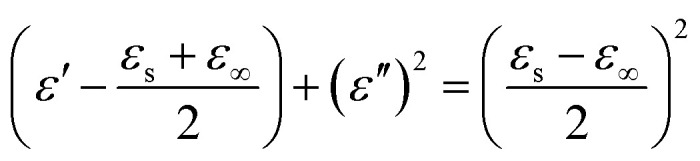


**Fig. 5:**
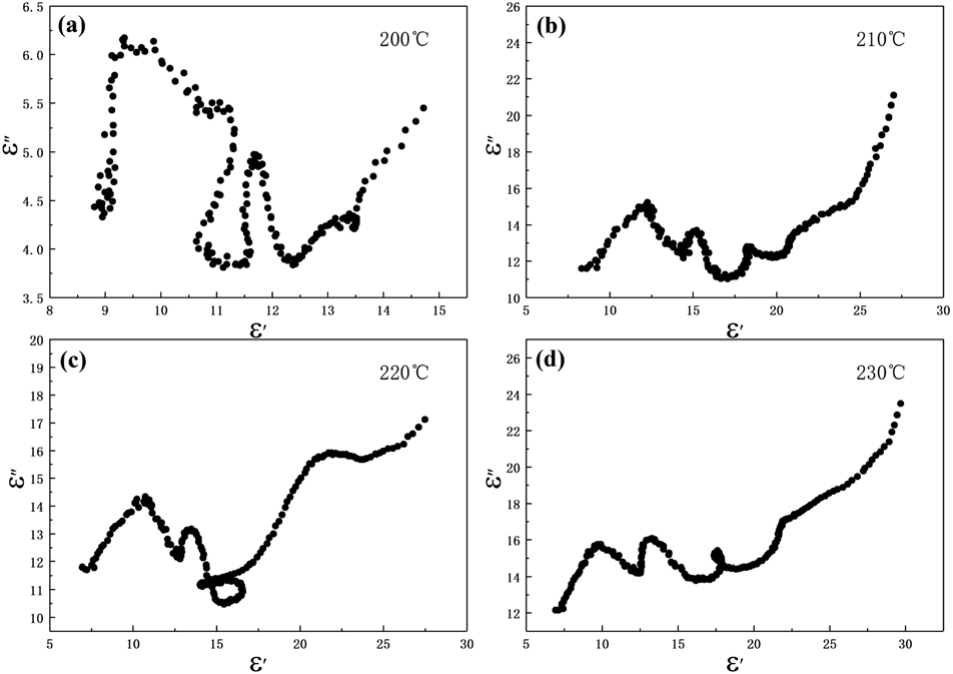
Cole–Cole semicircles for cobalt sulfide with 50% filling ratio under diffierent reaction temperature of (a) C1, (b) C2, (c) C3, (d) C4.

**Fig. 6:**
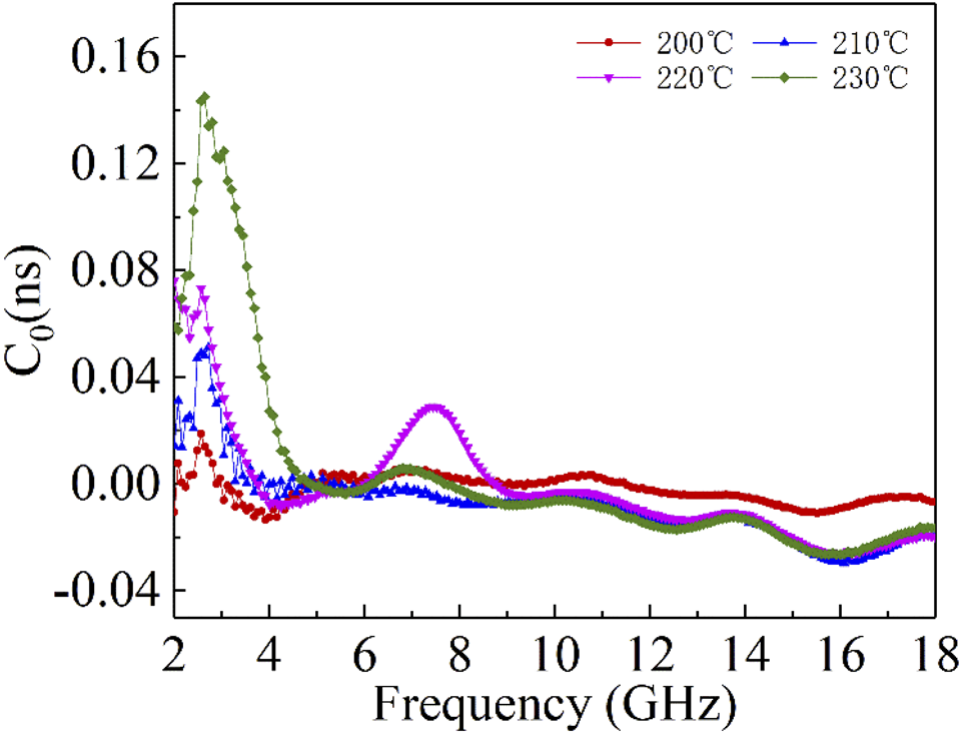
Constant *C*_0_ of cobalt sulfide with 50% filling ratio under diffierent reaction temperature.

In the above formula, *ε*_∞_ is the relative permittivity at the high frequency limit, *ε*_s_ is the static permittivity, *ε*_0_ is the dielectric constant under vacuum, *ω* is the angular frequency, *τ* is the relaxation time for polarization. Fig. 5(a)–(d) shows the Cole–Cole curves of C1 to C4 samples with 50% filling rate at different reaction temperatures. As shown in the figure, the Cole–Cole curves of the sample contains semicircles, representing a Debye relaxation process. It is interesting that the curves of the samples at different temperatures have similar Cole semicircles, which indicates that similar Debye relaxation processes occur between them. Also, the multiple dielectric relaxation process corresponds to the distinct polarization relaxation peak that can be observed in Fig. 4(b).^50^ This polarization relaxation phenomenon may mainly originate from interfacial polarization and dipole polarization. This polarization relaxation phenomenon may mainly originate from interfacial polarization and dipole polarization. The dipole polarization is mainly due to the intrinsic dipole and defective dipole of CoS and Co_9_S_8_ particles. According to the Maxwell–Wagner theory the difference in conductivity and permittivity of non-homogeneous particles under the condition of applied electric field will lead to the accumulation of space free charges at their interfaces, thus forming the interfacial polarization effect.^51^ This means that interfacial polarization occurs at the interface of CoS, Co_9_S_8_ and paraffin particles. Meanwhile, as shown in Fig. 4(a), the real part of the dielectric constant of all samples gradually decreases with the increase of frequency, may due to the effect of the weakening of the interfacial polarization caused by the gradual decrease of the accumulated charge at the interface. This suggests that interfacial polarization plays an important role in dielectric loss, which is consistent with the previously reported findings.^25^Fig. 4(c) and (d) show the trend of the real and imaginary parts of the magnetic permeability. After repeating the experiment to exclude testing errors, it was shown that the *μ*′′ of all samples became negative at high frequencies. It is clear that the imaginary part of the magnetic permeability represents the dissipation of magnetic energy, while a negative value means that magnetic energy is radiated from the particles. By Maxwell equation, the alternating electric field will produce a magnetic field, producing negative *μ*′′ and tan *δ*_m_ mainly due to the magnetic energy scattered by the magnetic field sample inherent magnetic loss can not offset these magnetic energy. This phenomenon has also occurred in previous studies of similar wave absorbing materials.^4,52,53^ It is known that the magnetic loss of absorbing materials mainly contains natural resonance, exchange resonance and eddy current loss.^2^ Meanwhile the eddy current effect can be calculated by using the following formula:^54^4
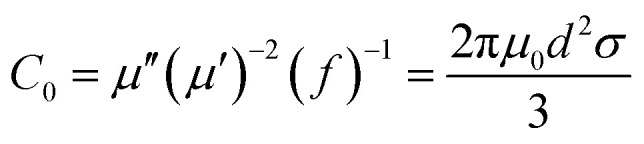
where *σ* and *μ*_0_ are corresponding to the electrical conductivity and the magnetic permeability in vacuum. As shown in Fig. 6, the *C*_0_ curves of the samples at different reaction temperatures have obvious fluctuations in the low frequency region, while they remain basically stable at high frequencies. This indicates that the magnetic loss in the high frequency (10–18 GHz) region of the sample is mainly due to the eddy current effect, while the magnetic loss in the low frequency (2–10 GHz) region is caused by the resonance effect. Moreover, the main source of the sample loss mechanism is the dielectric loss, and the magnetic loss has little effect.

The reflection loss of cobalt sulfide was fitted according to the above electromagnetic parameters. It is known that when the RL is less than −10 dB means that the absorbing material can absorb and attenuate 90% of the electromagnetic waves, which defined as the effective absorption bandwidth (EAD). According to transmission line theory, the RL can be expressed as:^55^5
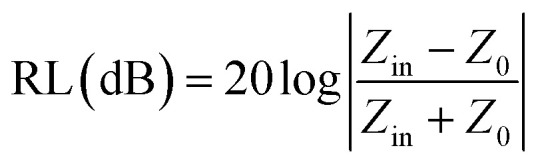
6
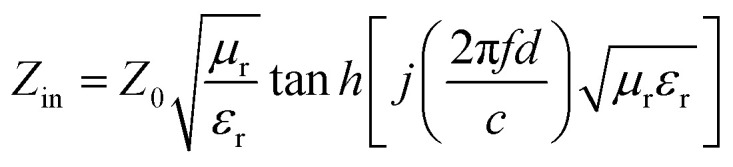
where *Z*_in_ is the input impedance of the absorber, *Z*_0_ is the characteristic impedance of free space, *μ*_r_ and *ε*_r_ are the complex permeability and complex dielectric constant, *f*, *c* and *d* is the frequency, the speed of light in free space and the thickness of the absorbing material, respectively.

**Fig. 7:**
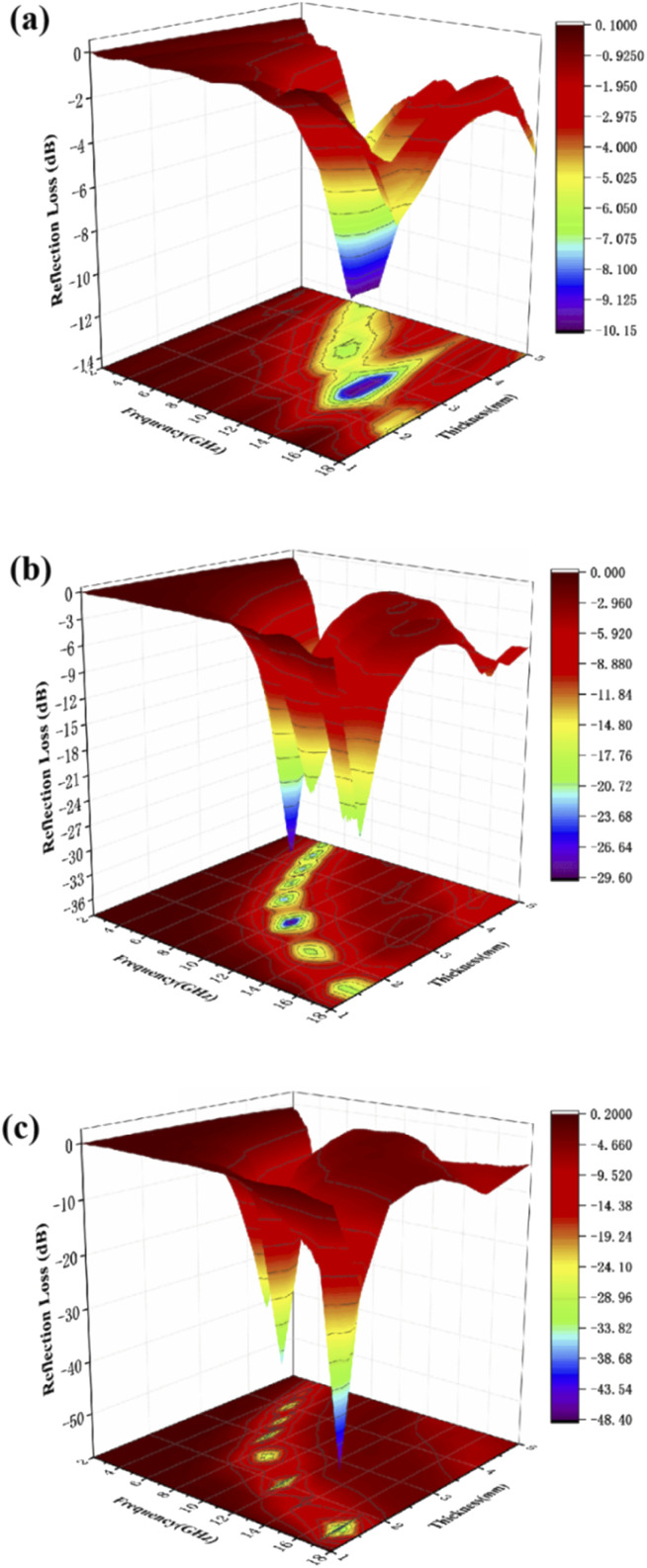
Three-dimensional plots of reflection loss values for paraffin composites of cobalt sulfide with 30% filling ratio of C1 (a), 40% filling ratio of C1 (b) and 50% filling ratio of C1 (c).

In order to optimize the thickness of the wave absorbing material, we tested the absorption performance of samples with 50%, 40% and 30% filling ratio by adjusting the sample filling ratio under the same conditions. As shown in Fig. 7(a) when the sample fill rate drops to 30%, the sample has no reflection loss less than −10 dB, which means the sample basically loses the absorbing performance. And when the sample filling rate decreases to 40% from Fig. 7(b), it shows a similar phenomenon as 50% from Fig. 7(c). The Fig. 7(b) shows that the RL_min_ for C1 is −29.7 dB at 10.4 GHz for a layer thickness of 2.5 mm, by adjusting the thickness of the absorbing sample from 1.5 mm to 5.0 mm, the effective absorption bandwidth can reach nearly 14.0 GHz for 4.0–18.0 GHz. By comparing the absorbing properties with those of the previous 50% samples, it can be found that the absorbing properties of the samples weakened accordingly as the filling rate decreased. This may be due to the incomplete local conductive network formed in the paraffin–cobalt sulfide complex system, and the samples accumulate charges at the interface equivalent to an isolated capacitor, relying only on the polarization effect and lacking sufficient dielectric loss for the modulation of the absorbing performance.

**Fig. 8:**
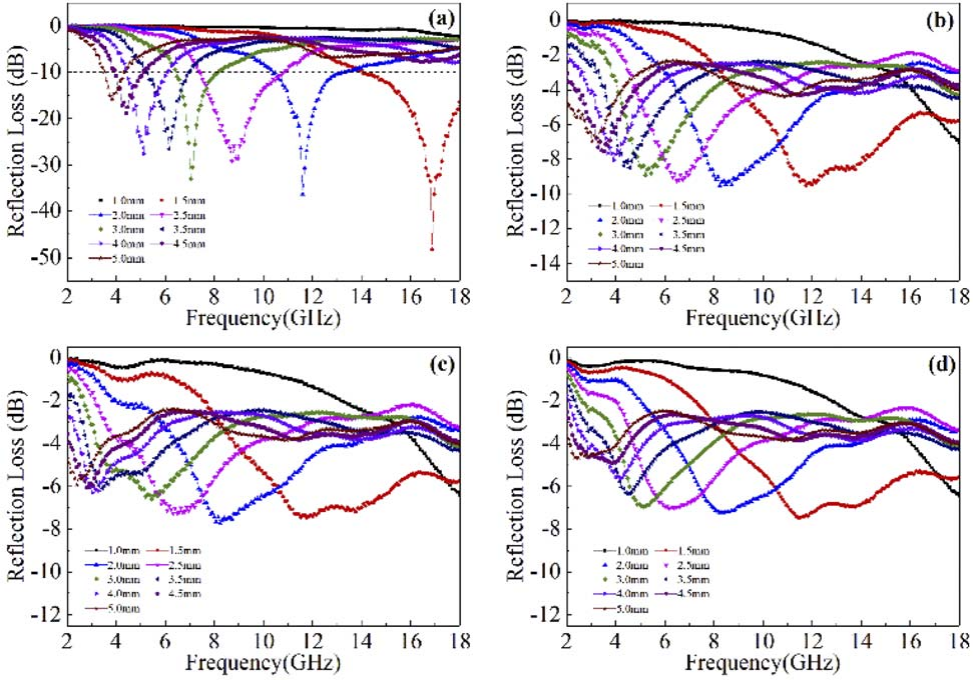
Reflection loss at different temperature and thicknesses for cobalt sulfide composites of C1 (a), C2 (b), C3 (c) and C4 (d) with filling ratios of 50%.

**Fig. 9:**
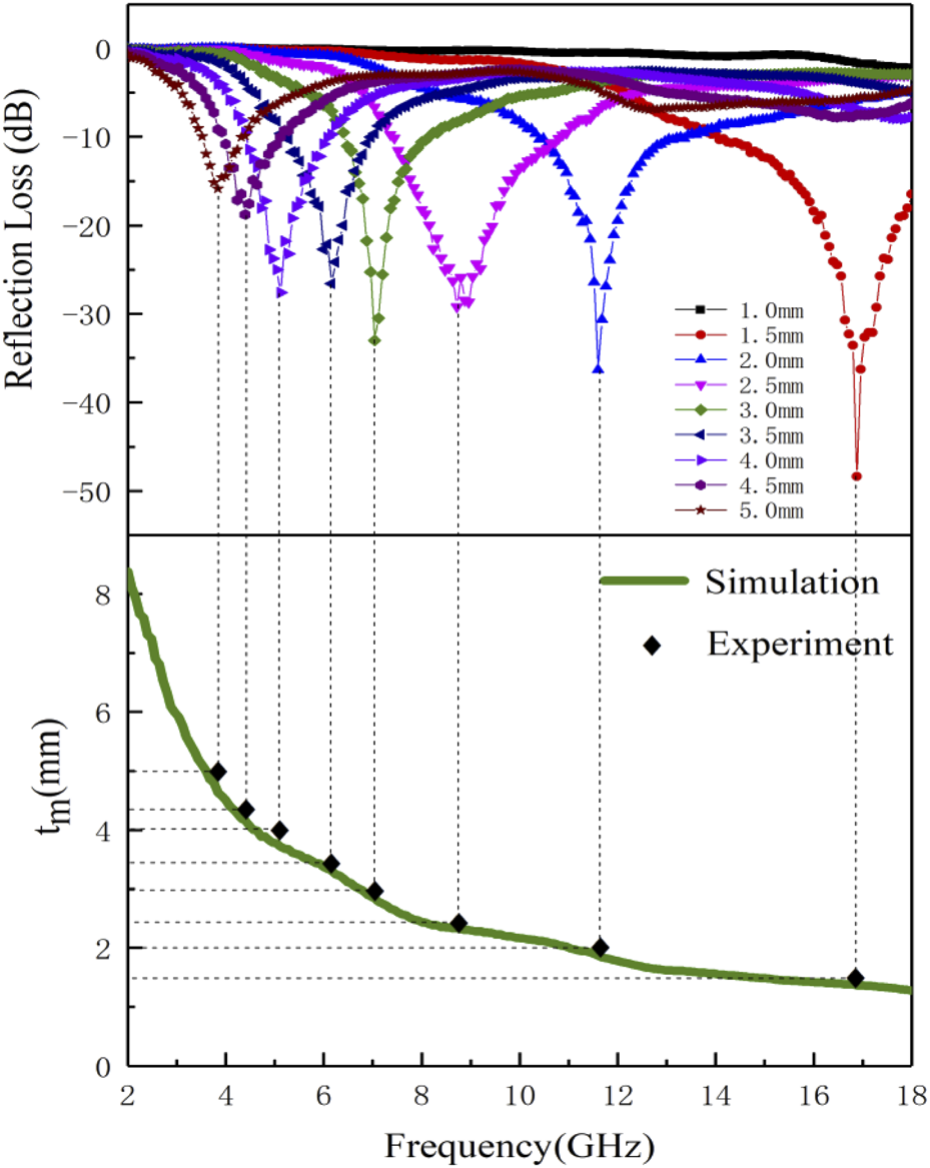
Corresponding matching thickness *vs.* frequency plots of the C1 with 50% filling ratio under *λ*/4 (the black dot represents experimental data and green line represents fitting plots).

Based on the determination of 50 wt% as the optimal absorption thickness of cobalt sulfide. The reflection loss of cobalt sulfide samples with 50% filling ratio in the thickness range of 1.0 mm to 5.0 mm at different reaction temperatures was obtained by calculation as shown in Fig. 8(a)–(d) which shows the RL of samples C1 to C4 corresponding to the reaction temperature 200 °C to 230 °C, respectively. As displayed in Fig. 8(a), the wave absorption performance of the cobalt sulfide is significantly improved when the temperature is increased to 200 °C. The RL_min_ for C2 is −48.4 dB at 16.8 GHz for a layer thickness of 1.5 mm corresponding to the ranges of effective absorption bandwidth is 13.7–18.0 GHz. By adjusting the thickness of the absorbing sample from 1.5 mm to 5.0 mm, an covering almost the entire measured frequency range of effective absorption bandwidth of 14.6 GHz can be obtained. Fig. 8(b)–(d) show that the wave absorption property of the cobalt sulfide sample becomes weaker with the further increase of the reaction temperature. This phenomenon is consistent with the data from previous tests, where the excessive dielectric loss of the sample led to a failure of impedance matching which results an imbalance between magnetic and dielectric losses. As shown in Fig. 9, almost all experimental points of the C1 sample with 50% fill rate match the *λ*/4 simulated curve, which this phenomenon can be explained by the quarter-wavelength cancellation model. As shown below:^56^7
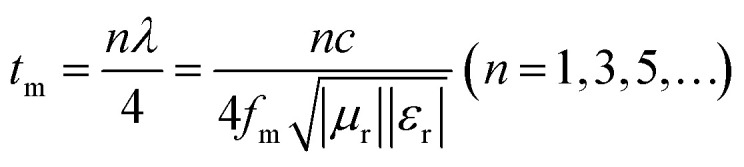
where *t*_m_ is the thickness of wave absorbing material, *λ* is the electromagnetic wave wavelength, *c* is the speed of electromagnetic waves and *f*_m_ is the peak frequency.

According to the impedance matching and attenuation characteristics, the mechanism of enhancing the microwave absorption performance can be discussed in detail. According to the propagation mechanism of the absorber, when electromagnetic waves are incident on the surface of the absorbing material, the first step is to meet the requirement that the electromagnetic waves can enter the inner part of the absorbing layer to the maximum extent. This requires the absorbing material to have a good impedance matching. According to the principle of impedance matching, when the value of |*Z*_in_/*Z*_0_| is close to 1, the incident electromagnetic wave is not reflected at the interface of the absorbing material, indicating good impedance matching. At the same time, the incident electromagnetic wave enters the inside of the medium without reflection, which means that the electromagnetic energy will be consumed by the absorber as much as possible. Meanwhile, the overall dissipation of the absorber can be characterized by the attenuation constant *α*, which can be calculated by the following equation:^57^8
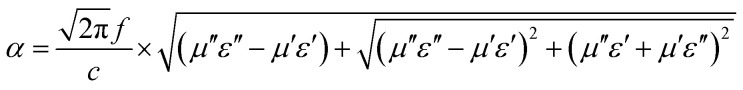


**Fig. 10:**
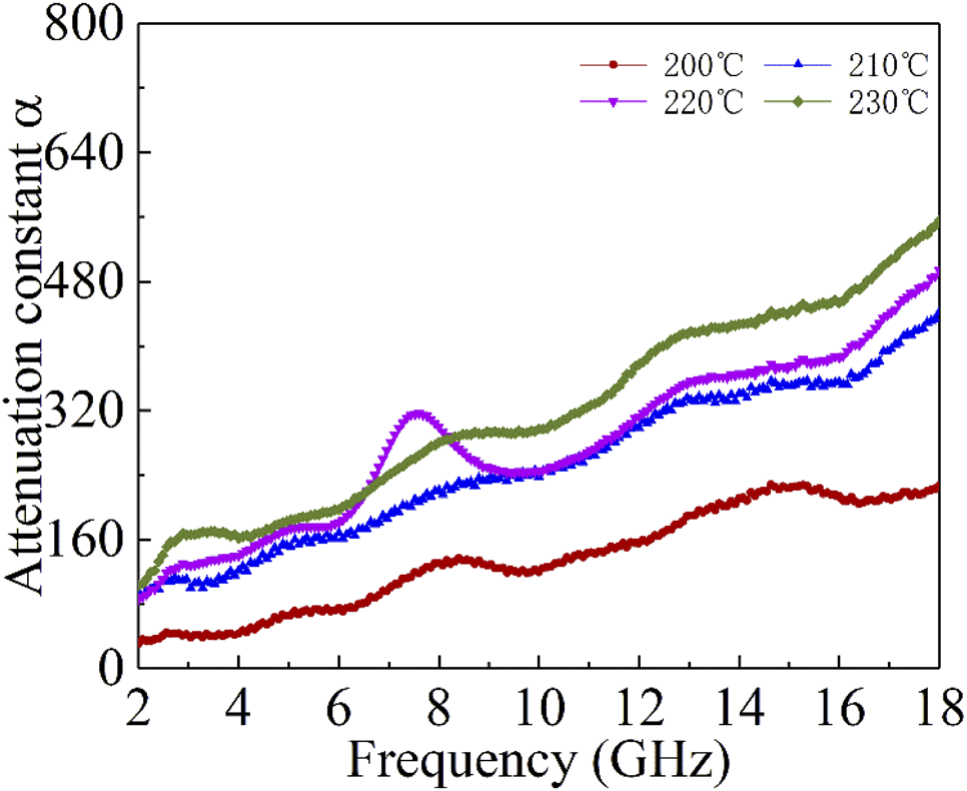
Attenuation constantα of cobalt sulfide with 50% filling ratio under diffierent reaction temperature.

Generally speaking, the large attenuation constant represents the strong attenuation ability of the sample, and the large values of *μ*′′ and *ε*′′ can also represent the strong attenuation ability as shown by the above formula. However, the attenuation capability of the absorber is regulated by both the impedance matching and the attenuation constant. Therefore, although the attenuation constants of the cobalt sulfide samples at other temperatures indicated in Fig. 10 are larger than the cobalt sulfide sample at 200 °C, the wave absorption performance is poor in comparison. This may be due to impedance matching, and we use the delta-function method (*Δ*) to characterize the degree of impedance matching, which can be expressed as:

9 Δ = |sin  *h*^2^(*Kfd*) − *M*|

**Fig. 11:**
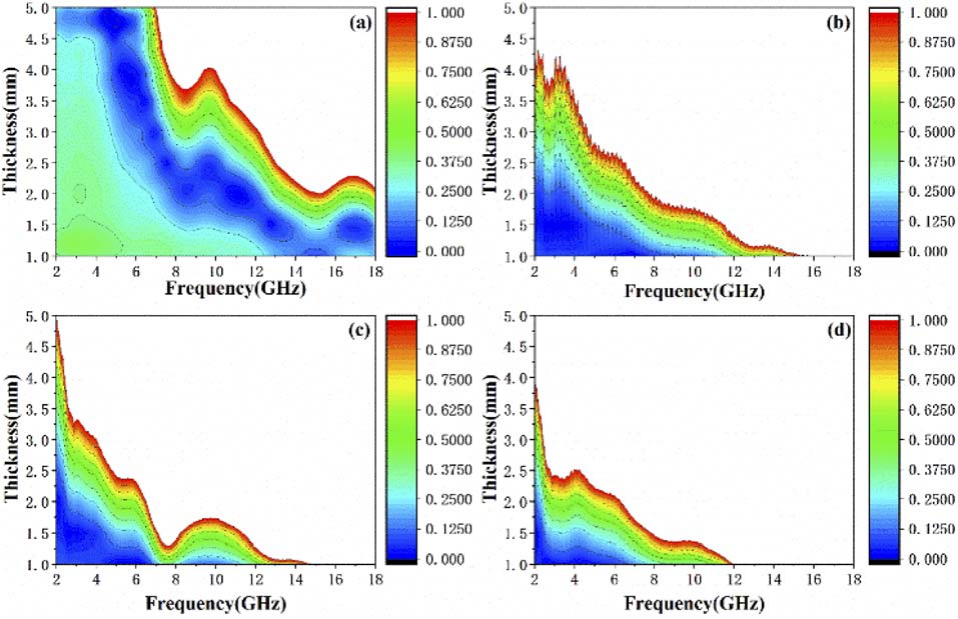
Calculated delta value maps for 50 wt% cobalt sulfide composites of C1 (a), C2 (b), C3 (c) and C4 (d) with various absorber thicknesses in 2.0–18.0 GHz.

10
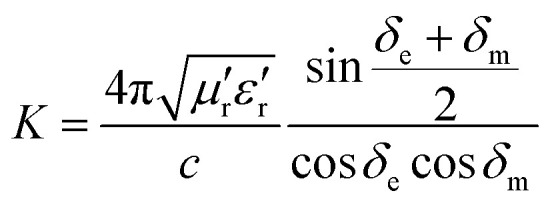
11
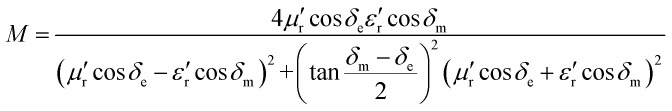
where *δ*_e_ and *δ*_m_ are corresponding to the dielectric loss tangent and magnetic loss tangent. As depicted in Fig. 11(a), the value of *Δ*. *Δ* tends to 0 represents a better impedance match than others from Fig. 9(b)–(d), while the opposite blank area indicates an impedance mismatch. Meanwhile, the heterogeneous cobalt sulfide particle changes its two-phase ratio as the reaction temperature increases, which may lead to an imbalance of magnetic and dielectric losses making impedance mismatch. Excellent impedance matching and attenuation constants are jointly regulated, making the sample of C1 with 50% filling ratio have better absorption performance than others.

The excellent wave absorption performance of cobalt sulfide may be related to its highly efficient absorption mechanism. The unique three-dimensional nanostructure, rich surface and easy generation of hollow structure make it difficult to transmit or reflect electromagnetic waves after incident, and most of them are converted into heat to be attenuated. In addition, with the adjustment of reaction temperature, the impedance matching and attenuation ability of heterophase cobalt sulfide are improved. These mechanisms enable cobalt sulfide to possess excellent interfacial polarization, dipole polarization and dielectric loss capabilities, co-modulating its electromagnetic energy absorption capacity.

## Conclusions

In summary, a heterophase sample of cobalt sulfide at different reaction temperatures was synthesized by a simple hydrothermal method. Its microstructural composition, electromagnetic parameters and wave absorption properties were investigated. With the increase of reaction temperature, the wave absorption properties of cobalt sulfide showed a weakening trend under the joint regulation of impedance matching and attenuation characteristics, and the microwave absorption performance matched with the quarter-wavelength model well. The RL_min_ for cobalt sulfide with reaction temperature of 200 °C and 50% filling ratio is −48.4 dB at 16.8 GHz for a layer thickness of 1.5 mm corresponding to the ranges of effective absorption bandwidth is 4.3 GHz which almost covers the whole Ku band. Meanwhile, by adjusting the thickness of the absorbing sample from 1.5 mm to 5.0 mm, an covering almost the entire measured frequency range of effective absorption bandwidth of 14.6 GHz can be obtained. These findings indicate that cobalt sulfide heterophase absorbing materials at different reaction temperatures have the advantages of thin thickness, light weight, wide effective absorption bandwidth, low cost and simple synthesis method, which is a very promising microwave absorbing material.

## Conflicts of interest

There are no conflicts to declare.

## Supplementary Material
